# Defining the global health system and systematically mapping its network of actors

**DOI:** 10.1186/s12992-018-0340-2

**Published:** 2018-04-17

**Authors:** Steven J. Hoffman, Clarke B. Cole

**Affiliations:** 1 Global Strategy Lab, Dahdaleh Institute for Global Health Research, Faculty of Health and Osgoode Hall Law School, York University, 4700 Keele Street, Toronto, Ontario M3J 1P3 Canada; 2 Department of Health Research Methods, Evidence & Impact and McMaster Health Forum, McMaster University, Hamilton, Ontario Canada; 3 Department of Global Health & Population, Harvard T.H. Chan School of Public Health, Harvard University, Boston, MA USA; 4 Access to Medicine Foundation, Amsterdam, The Netherlands

**Keywords:** Global Health, Internet, Search engine, World Health Organization

## Abstract

**Background:**

The global health system has faced significant expansion over the past few decades, including continued increase in both the number and diversity of actors operating within it. However, without a stronger understanding of what the global health system encompasses, coordination of actors and resources to address today’s global health challenges will not be possible.

**Methods:**

This study presents a conceptually sound and operational definition of the global health system. Importantly, this definition can be applied in practice to facilitate analysis of the system. The study tested the analytical helpfulness of this definition through a network mapping exercise, whereby the interconnected nature of websites representing actors in the global health system was studied.

**Results:**

Using a systematic methodology and related search functions, 203 global health actors were identified, representing the largest and most transparent list of its kind to date. Identified global health actors were characterized and the structure of their social network revealed intriguing patterns in relationships among actors.

**Conclusions:**

These findings provide a foundation for future inquiries into the global health system’s structure and dynamics that are critical if we are to better coordinate system activities and ensure successful response to our most pressing global health challenges.

**Electronic supplementary material:**

The online version of this article (10.1186/s12992-018-0340-2) contains supplementary material, which is available to authorized users.

## Background

The global health system has faced significant expansion over the past few decades, including continued increase in both the number and diversity of actors within it [1–5]. Recognition is growing that better coordination of these actors is necessary if we are to ensure effective response to the most pressing contemporary global health challenges. However, the research literature demonstrates little agreement over definitions of relevant concepts and their practical applications [6]. Without a stronger empirical understanding of what the global health system actually encompasses – including its components, how they operate, and how well they do so – such coordination will be more difficult.

In particular, there is a lack of understanding over which actors should actually be considered as part of the global health system. The range of actors included is frequently cited at “more than 40 bilateral donors, 26 UN agencies, 20 global and regional funds and 90 global health initiatives” [7]. However, given the continued expansion of the system, this 2007 statistic is outdated and information is also lacking on how it was derived and which actors it includes. In order to develop an updated account of global health actors, we must find a meaningful way to map them, considering not only their existence but also the ways in which they interact with one another. Online networks – constructed by real-world actors – present a unique solution to this challenge. Coscia, Hausmann & Hidalgo (2013) used online information to study the structure of international aid coordination, creating and mapping a network of donor organizations, recipient countries, and development issues [8]. Similarly, Coscia & Rios (2012) developed and validated a framework that used Internet content, such as online newspapers and blogs, to reveal areas of operation of Mexican drug trafficking organizations [9]. These studies demonstrated the potential to use online networks for global health system analysis. Further studies using offline data demonstrate the value of mapping social networks within the health sector. For example, Bowen et al. (2014) applied social network analysis to identify key organizations engaged in developing health-related climate change adaptation activities in Cambodia [10].

This study aims to advance our understanding of the global health system by answering two questions. First, what is the ‘global health system’? And second, who populates this system? While many answers are possible, we limit ourselves to definitions that can be operationalized with inclusion/exclusion criteria and mapping methods that are systematic, transparent, and replicable. In this way, we produce work that is hopefully analytically useful, minimally biased, and foundational for future inquiries.

In answering these two questions, we first present an operational definition of the global health system that sets clear boundaries and can actually be applied to map global health actors and their relationships. Second, we used online network relationships to generate a list of 203 global health actors and characterize those actors; in effect, using the online network of global health actors to better understand the offline global health system [11].

## Methods

### Defining the global health system

Relevant literature from global health, international relations, law, political science, and public policy was reviewed to understand how scholars are conceptualizing the global health system and how cognate global systems have previously been defined. This literature was used to inform development of a clear, applicable definition of the global health system (see Additional file 1 for a more detailed account of this process).

### Mapping the global health system

The helpfulness of this definition was tested through application to a mapping exercise of the global health system, in which websites that represent global health actors were identified. Using a systematic search protocol, a network of global health actors was created, characterized, and its social network analyzed. This methodology took advantage of the Internet’s network structure in a novel way, mapping online interactions to probabilistically identify key offline relationships in the global health system.

Importantly, the methodology was designed to be internally valid, transparent, and replicable, identifying a minimally-biased network of actors operating within the global health system. It was not designed to create a comprehensive or complete list of all actors operating within the global health system or to identify a list of actors based on their power or influence within the system. Through this process, it is hoped that our study output will provide a snapshot of the current global health architecture, reflecting a true network that can be used to both inform our understanding of the global health system and allow for further application and development of our methodology.

#### Data mining

A systematic search of the Internet was conducted to identify the online network of global health actors. This involved the use of a *related search function* that can identify websites that represent global health actors. Related search functions use algorithms – different combinations of connectivity analysis, content analysis and page usage – to identify web pages that are topically similar but not identical to one another. Web connectivity algorithms, such as co-citation analysis, exploit the hyperlink-structure of the Internet to find web pages that reference each other. They work under the basic assumption that web pages connected by hyperlinks contain related content and, if the pages have distinct authors, this linkage suggests the creator of one website found the content of referenced web pages to be valuable. Content analysis algorithms evaluate similarities in topical content found across web pages. This means that web pages with information on similar topics will be considered related. Page usage relates to information gained about a web page when search engine users select a particular link following a given query (e.g., if a user searches for ‘university’, and selects the search result ‘Harvard’, it logically follows that Harvard is likely a university) [12, 13].

Accordingly, this methodology assumes a website can serve as a minimum criterion for including an actor in a preliminary list of global health actors, as most actors with a capacity to influence global health will, at a minimum, have an online presence. A related search function was used to identify global health actors under the assumption that actors operating offline in the global health system are likely to reference other actors they consider relevant and valuable through linkages to those actors’ websites on their own website. It also assumes global health actors are likely to include similar topical content on their websites, and that Internet users are likely to follow similar search and retrieval patterns in accessing the sites of different global health actors.

Searches were conducted using the publicly available Google search engine (www.google.com) and its corresponding crawler, Googlebot, which maintains a real-time index of over 100 million gigabytes [14]. Searches used the “related:URL” query refinement for which the user enters a specific known URL to find websites that link to it, share similar content and attract the same users [15]. A proxy server was used to search anonymously from a United States–based (U.S.) IP address, and all browser history, caches, and cookies were cleared to prevent personalized results. A pilot test was conducted to refine the systematic search methodology and inclusion/exclusion criteria (detailed more thoroughly in Additional file 2).

A snowball sampling search process was started with the World Health Organization’s (WHO’s) website, thereby placing this United Nations (UN) agency at the centre of our global health system mapping [3, 4]. This was done by conducting a related search using its website (i.e., “related:www.who.int”). Next, in a second-stage search, related searches were conducted on all eligible websites identified in the original search. Results were extracted for eligibility screening.

#### Data screening

Websites retrieved through the review were included in our mapping of the global health system if they met the following three criteria, based on our definition for a global health actor (see Additional files 3 and 4 for detailed screening forms):

1. The result represents an individual or organization (i.e., an actor).
2. The actor operates in three or more countries (i.e., transnationally).
3. The actor identifies improving health as one of its primary intents (i.e., health focus).

The inclusion/exclusion criteria were applied in two stages. In the first stage, website summaries available in search results were reviewed to determine whether the sites met at least two of the criteria. Those websites that passed stage 1 screening were then reviewed in full to determine whether they met all three criteria. If so, they were included in the final mapping.

**Fig. 1:**
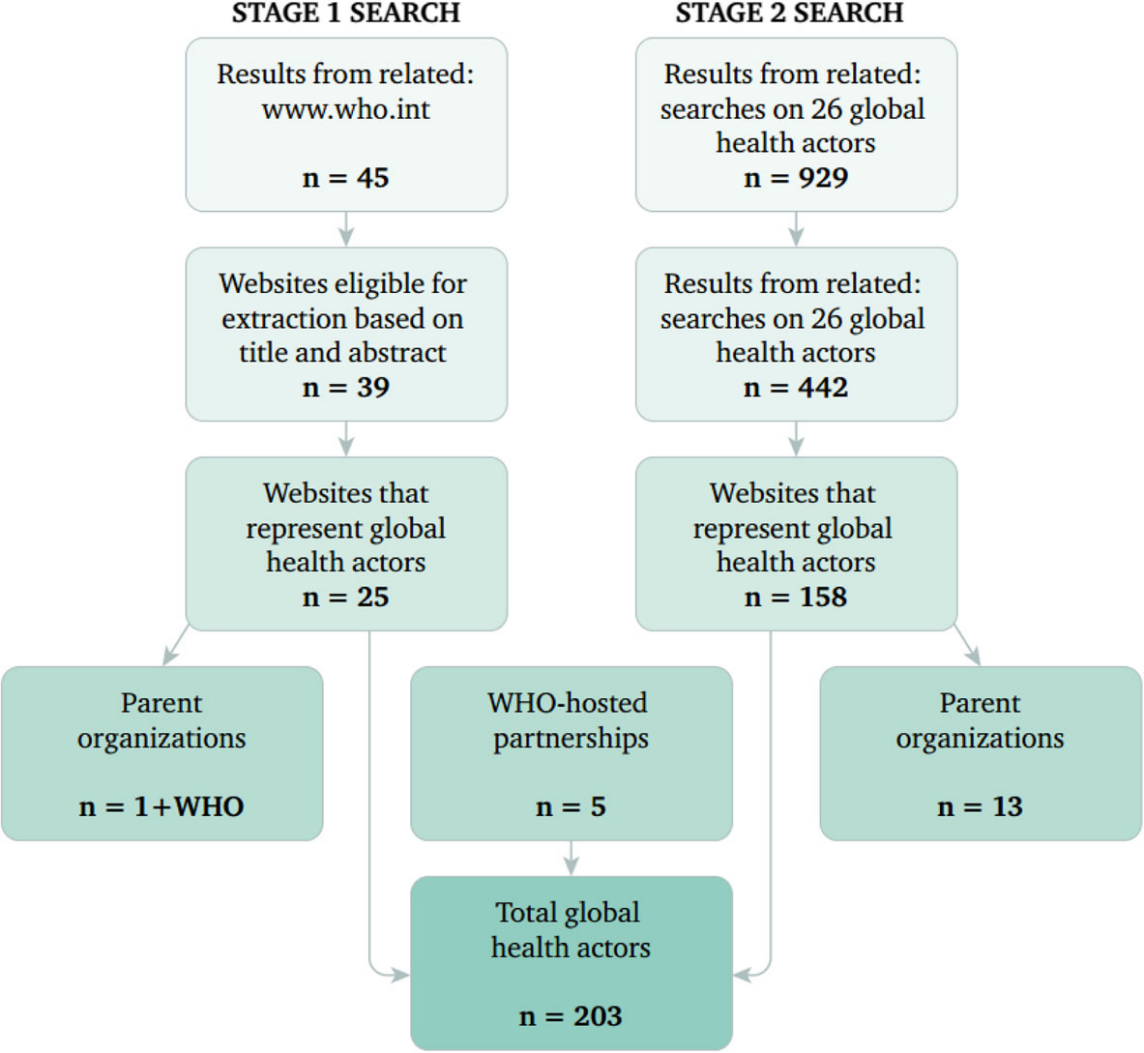
Flowchart of the systematic review

Specifically, for the full website review, the “About” web page (or its equivalent) of each retrieved website was reviewed for its ability to meet all three inclusion criteria and eligible websites were included as global health actors in the systematic review. All uncertainties were reviewed and, where necessary, additional web pages on an actor’s website were accessed for further information. Identified parent organizations were evaluated for inclusion in the review. If eligible, corresponding child organizations were omitted (see Fig. 1 for a flowchart of the review process). A *parent* organization is the umbrella organization in cases where there are multiple arms or affiliates as part of a single organization. A *child* organization is one autonomous section or offshoot of a parent organization. For example, CARE International is a parent organization and CARE Canada is one of its child organizations. If both organizations met the inclusion criteria during the review, CARE International would be included in the final review and CARE Canada would be omitted.

#### Data analysis

Findings from the systematic review were entered into a database of actors in the global health system. To characterize each global health actor, additional data were extracted from its website. Global health actors were categorized according to primary types of entities in the global health system as outlined in Frenk & Moon (2013) [3]: headquarters location, year of inception, and whether the primary intent of the organization was to improve health (as opposed to whether promoting health was just one of several primary objectives). Actors were placed into one distinct category under each variable.

The network was modelled to allow visualization of the global health system and facilitate observations of the network structure. The network was constructed such that each global health actor was assigned a node and characteristics of each respective actor were recorded as node attributes. Directed edges were used to indicate each time a particular actor linked to another actor in the network (i.e., each time the “related:URL” search for a global health actor’s website yielded a result that was also an actor). All duplicate results were included such that a directed edge exists in the network to indicate every time an actor appeared in any set of “related:URL” search results throughout the review. Gephi 0.8.2 beta was used to construct and analyze the network [16].

According to social network theory, actors gain power within social networks by holding advantageous positions relative to other actors. Centrality, one of the most studied metrics in social network analysis, allows an actor’s position in its network to be described relative to others’. Three relatively simple centrality metrics that are important and popularly used were applied in this analysis: degree centrality, betweenness centrality and closeness centrality [17, 18].

Nodes with high degree centrality are connected to a large number of other nodes in the network. They have a high probability of receiving and transmitting information that flows through the network, and are therefore considered highly active players. Betweenness centrality measures how often a node appears within the shortest path between nodes. It describes an actor’s level of control over information in the network, and as such, actors with high betweenness centrality function as information brokers in the network relied upon to communicate and enhance collaboration between sub-communities. Closeness centrality measures the degree to which a node is close to all others in the network. Nodes that are central by closeness can reach most or all others in the network on average in fewer steps than others. They are described as being able to communicate with high efficiency in terms of time and cost. Analysis of the global heath network using these three metrics allows global health actors with three basic sources of advantage in the network to be identified: advantage based on communication activity, control of information, and independence and efficiency, respectively [17, 18].

### Validation exercise

The findings of this study were presented to senior leaders of seven prominent global health organizations (i.e., Gavi, the Vaccine Alliance, Global Fund to Fight AIDS, Tuberculosis and Malaria, Joint United Nations Programme on HIV/AIDS [UNAIDS], United Nations Population Fund, United Nations Children’s Fund [UNICEF], WHO and World Bank) at a workshop in Geneva, Switzerland in December 2014 to assess the analytic helpfulness and comprehensiveness of the findings.

## Results

### Defining the global health system

The literature review identified an abundance of definitions for key terms related to the global health system (see Additional file 1 for detailed methodology and findings of the literature review). Terms were defined using a variety of approaches and with varying boundaries, highlighting the usefulness of conceptualizing the global health system in different ways to serve different purposes [3].

Building on the work of Slzezak et al. (2010), [4] Hoffman et al. (2012), [19] and Frenk & Moon (2013), [3] the following definition for the global health system is proposed:

The *global health system* includes the *transnational* actors that have a primary intent to improve health and the *polylateral arrangements* for *governance*, *finance,* and *delivery* within which these actors operate.

Under this definition, the interactions between global health actors are influenced by the actors themselves, the internal arrangements of the system, and external forces, such as actors and arrangements from other important global policy domains. Accordingly, a *global health actor* is defined as an individual or organization that operates transnationally with a primary intent to improve health (see Table 1 for further explanation of key terms found within this definition).

**Table 1:** Global health system definition

Transnational actors: Individuals or organizations that operate in a way that transcends national political borders. *Unlike the term international, which may require actors to be stationed in multiple countries, transnational actors may be stationed in only one country so long as they operate across borders.*
Polylateral: The interactions among, and governance of, states and non-state actors, which includes interactions between states, between non-state actors, and between states and non-state actors [26]. *While the term bilateral concerns relations between two states and the term multilateral concerns relations between three or more states, the term polylateral is more inclusive in that it also considers interactions of non-state entities* [26].
Global health system arrangements include: [27]
Delivery arrangements: relate to how health services are delivered, accessed and catered to meet local priorities, and focus on factors that determine how care is designed to meet consumers’ needs, by whom care is provided, where care is provided and with the supports used to those providing and receiving care.
Financial arrangements: relate to how finances flow through health systems, and focus on how systems are financed, types of funding organizations, how to remunerate providers, how products and services are purchased and the incentive structures for consumers.
Governance arrangements: relate to how a health system is governed, and focus on issues such as policy authority, organizational authority, commercial authority, professional authority and about how stakeholders are involved in health systems decisions and on what terms.

The definition developed is holistic in nature, whereby the system is viewed from a global perspective. As a result, emphasis is placed on transnational actors engaged in such matters as protecting health security, promoting human rights, responding to humanitarian crises, and facilitating international development, and not on national actors or any particular sub-system of actors. The definition is comprehensive in its ability to consider multiple aspects of the system. First, the definition is inclusive of actors that *operate* with a primary intent to improve health, not limiting the system to include only those actors that hold powerful or influential roles in the global health system. Additionally, the definition considers relations between actors, arrangements that influence the system’s functions, as well as the interaction of the system components with internal and external forces. Importantly, the definition also applies to developing our practical understanding of the global health system. The ability to effectively translate conceptual aspects of the definition into clear inclusion/exclusion criteria for a systematic review process, as outlined in this study, illustrates is value at the operational level (refer to Additional files 3 and 4 for detailed application of the definition to inclusion/exclusion screening criteria).

### Mapping the global health system

#### Related search results

A first-stage search for “related:www.who.int” was conducted on March 14, 2014, yielding 45 actors. In total, 25 of these actors plus one parent organization met the inclusion criteria to be considered global health actors. Subsequently, the home page URLs of each of these actors’ websites were used in a second-stage “related:URL” search on March 17, 2014. The one exception was the “related:plan-international.org” search, which was conducted on March 24, 2014. A total of 26 independent second-stage searches were conducted and 929 results retrieved. 572 unique results were reviewed. 158 websites, plus those of 13 parent organizations, met the inclusion criteria in this second-stage search to be considered representative of global health actors.

In addition, five WHO-hosted partnerships were included as global health actors [20]. Four of these WHO-hosted partnerships would have been impossible to find through our Internet-based network mapping given their websites are hosted on WHO’s website. These include the Alliance for Health Policy and Systems Research, European Observatory on Health Systems & Policies, Global Health Workforce Alliance, and Partnership for Maternal, Newborn & Child Health. The International Drug Purchase Facility (UNITAID) was also included because it was the only remaining WHO-hosted partnership as listed in provisional agenda item 11.4 of the 134th session of the WHO Executive Board, *Hosted Health Partnerships*, which neither has a WHO-hosted website nor was identified in the “related:URL” Internet searches*.*

When including WHO as the seed global health actor, in total we identified 203 global health actors (see Table 2 for a list of global health actors, their corresponding websites and data on selected characteristics).

**Table 2:** List of global health actors included in the systematic review

Actor		Code	URL	Type	Headquarters Location		Health as primary intent?	Year of Inception
1	Abt Associates	ABA	abtassociates.com	7	Cambridge, MA	USA	No	1965
2	Accordia Global Health Foundation	AGH	accordiafoundation.org	6	Washington, DC	USA	Yes	2000
3	ACTION (Global Health Advocacy Partnership)	ACT	action.org	6	Washington, DC	USA	Yes	2004
4	Action Against Hunger International	AAH	actionagainsthunger.org	6	New York City, NY	USA	Yes	1979
5	Action on Smoking and Health	ASH	ash.org	6	Washington, DC	USA	Yes	1967
6	Advocates for Youth	AFY	advocatesforyouth.org	6	Washington, DC	USA	Yes	1980
7	Aeras	AER	aeras.org	6	Rockville, MD	USA	Yes	2003
8	Africa Fighting Malaria	AFM	fightingmalaria.org	6	Durban	South Africa	Yes	2000
9	African Leaders Malaria Alliance	ALM	alma2015.org	2	New York City, NY	USA	Yes	2009
10	Africare	AFR	africare.org	6	Washington, DC	USA	No	1970
11	Against Malaria Foundation	AMF	againstmalaria.com	6	St. Albans	UK	Yes	2004
12	AIDS Healthcare Foundation	AHF	aidshealth.org	6	Amsterdam	Netherlands	Yes	1987
13	Alliance for Health Policy and Systems Research	AHP	who.int/alliance-hpsr	4	Geneva	Switzerland	Yes	1999
14	America Association of Occupational Health Nurses	AAO	aaohn.org	8	Pensacola, FL	USA	No	1998
15	American Association of Veterinary Parasitologists	AAV	aavp.org	8	Shawnee, KS	USA	No	1956
16	American College of Preventive Medicine	ACP	acpm.org	8	Washington, DC	USA	Yes	1954
17	American International Health Alliance	AIH	aiha.com	6	Washington, DC	USA	Yes	1992
18	American Jewish World Service	AJW	ajws.org	6	New York City, NY	USA	No	1985
19	American Public Health Association	APH	apha.org	8	Washington, DC	USA	Yes	1872
20	American Red Cross	ARC	redcross.org	6	Washington, DC	USA	No	1881
21	American Refugee Committee	ARO	arcrelief.org	6	Minneapolis, MN	USA	No	1979
22	American Society for Microbiology	ASM	asm.org	8	Washington, DC	USA	No	1899
23	American Society of Tropical Medicine and Hygiene	AST	astmh.org	6	Deerfield, IL	USA	Yes	1903
24	American Thoracic Society	ATS	thoracic.org	8	New York City, NY	USA	Yes	1905
25	amfAR (Foundation for AIDS Research)	AMA	amfar.org	6	New York City, NY	USA	Yes	1985
26	Anaerobe Society of the Americas	ASA	anaerobe.org	8	Los Angeles, CA	USA	Yes	1992
27	Asia Pacific Malaria Elimination Network	APM	apmen.org	4	Herston	Australia	Yes	2009
28	Association of Public Health Laboratories	APL	aphl.org	6	Silver Spring, MD	USA	Yes	1951
29	Australasian College of Tropical Medicine	ACM	tropmed.org	8	Brisbane	Australia	Yes	1991
30	AVAC: Global Advocacy for HIV Prevention	AVA	avac.org	6	New York City, NY	USA	Yes	1995
31	AVERT	AVE	avert.org	6	Horsham	UK	Yes	1986
32	Bill & Melinda Gates Foundation	BMG	gatesfoundation.org	5	Seattle, WA	USA	No	2000
33	BIO Ventures for Global Health	BVG	bvgh.org	6	Seattle, WA	USA	Yes	2004
34	Campaign for Tobacco-Free Kids	CTF	global.tobaccofreekids.org	6	Washington, DC	USA	Yes	1995
35	CARE International	CAI	care-international.org	6	Geneva	Switzerland	No	1945
36	Caritas International	CRI	caritas.org	6	Vatican City	Vatican City State	No	1897
37	Catholic Medical Mission Board	CMM	cmmb.org	6	New York City, NY	USA	Yes	1912
38	Catholics for Choice	CFC	catholicsforchoice.org	6	Washington, DC	USA	No	1973
39	CDC Foundation	CDC	cdcfoundation.org	6	Atlanta, GA	USA	Yes	1995
40	Center for Global Development	CGD	cgdev.org	6	Washington, DC	USA	No	2001
41	Center for Health and Gender Equity	CHG	genderhealth.org	6	Washington, DC	USA	No	1994
42	Center for International Environmental Law	CIE	ciel.org	6	Washington, DC	USA	No	1989
43	Center for Reproductive Rights	CRR	reproductiverights.org	6	New York City, NY	USA	No	1992
44	Centers for Disease Control and Prevention	CDP	cdc.gov	1	Atlanta, GA	USA	Yes	1946
45	Chemonics International	CHI	chemonics.com	7	Washington, DC	USA	No	1975
46	Christian Connections for International Health	CCI	ccih.org	6	McLean, VA	USA	Yes	1987
47	CONRAD	CON	conrad.org	6	Arlington, VA	USA	Yes	1986
48	Consultative Group on Early Childhood Care and Development	CGE	ecdgroup.com	6	Toronto	Canada	No	1984
49	CORE Group	COG	coregroup.org	6	Washington, DC	USA	Yes	1997
50	Countdown to 2015	COT	countdown2015mnch.org	6	Geneva	Switzerland	Yes	2005
51	Direct Relief	DIR	directrelief.org	6	Santa Barbara, CA	USA	Yes	1948
52	Doctors for Global Health	DGH	dghonline.org	6	Decatur, GA	USA	No	1995
53	Elizabeth Glaser Pediatric AIDS Foundation	EGP	pedaids.org	6	Washington, DC	USA	Yes	1988
54	Elton John AIDS Foundation	EJA	ejaf.org	6	London	UK	Yes	1992
55	EngenderHealth	ENH	engenderhealth.org	6	New York City, NY	USA	Yes	1943
56	Episcopal Relief & Development	ERD	episcopalrelief.org	6	New York City, NY	USA	No	1940
57	European & Developing Countries Clinical Trials Partnership	EDC	edctp.org	2	The Hague	Netherlands	Yes	2003
58	European AIDS Treatment Group	EAT	eatg.org	6	Brussels	Belgium	Yes	1992
59	European Food Information Council	EFI	eufic.org	6	Brussels	Belgium	No	1995
60	European Generic Medicines Association	EGM	egagenerics.com	7	Brussels	Belgium	No	1993
61	European Medical Students’ Association	EMS	emsa-europe.org	6	Brussels	Belgium	No	1991
62	European NGOs for Sexual and Reproductive Health and Rights, Population and Development	ENS	eurongos.org	6	Brussels	Belgium	Yes	1996
63	European Observatory on Health Systems and Policies	EOH	euro.who.int/en/about-us/partners/observatory	4	Brussels	Belgium	Yes	1998
64	European Respiratory Society	ERS	ersnet.org	8	Lausanne	Switzerland	Yes	1990
65	European Vaccine Initiative	EVI	euvaccine.eu	6	Heidelberg	Germany	Yes	1998
66	Family Care International	FCI	familycareintl.org	6	New York City, NY	USA	Yes	1986
67	Federation of American Societies for Experimental Biology	FAS	faseb.org	8	Bethesda, OH	USA	Yes	1912
68	Feed the Future	FTF	feedthefuture.gov	1	Washington, DC	USA	No	2010
69	FHI 360 (formerly Family Health International)	FHI	fhi360.org	6	Durham, NC	USA	No	2011
70	Firelight Foundation	FIF	firelightfoundation.org	6	Santa Cruz, CA	USA	No	2000
71	Fistula Foundation	FSF	fistulafoundation.org	6	San Jose, CA	USA	Yes	2000
72	Food and Agriculture Organization of the United Nations	FAO	fao.org	2	Rome	Italy	No	1945
73	Foundation for Innovative New Diagnostics	FIN	finddiagnostics.org	6	Geneva	Switzerland	Yes	2003
74	Foundation for International Medical Relief of Children	FIM	fimrc.org	6	Philadelphia, PA	USA	Yes	2002
75	Framework Convention Alliance for Tobacco Control	FCA	fctc.org	6	Geneva	Switzerland	No	1999
76	Futures Group	FUG	futuresgroup.com	7	Washington, DC	USA	Yes	1971
77	Gavi, the Vaccine Alliance	GAA	gavialliance.org	4	Geneva	Switzerland	Yes	1999
78	GBCHealth	GBH	gbchealth.org	6	New York City, NY	USA	Yes	2001
79	Global Advisors Smokefree Policy	GAS	njgasp.org	6	Summit, NJ	USA	No	1974
80	Global Alliance for TB Drug Development	GAT	tballiance.org	4	New York City, NY	USA	Yes	2000
81	Global Coalition Against Child Pneumonia	GCA	worldpneumoniaday.org	4	Baltimore, MD	USA	Yes	2009
82	Global Communities	GLC	globalcommunities.org	6	Silver Spring, MD	USA	No	1952
83	Global Health Corps	GHC	ghcorps.org	6	New York City, NY	USA	No	2008
84	Global Health Council	GHO	globalhealth.org	6	Washington, DC	USA	Yes	1972
85	Global Health Workforce Alliance	GHW	who.int/workforcealliance	4	Geneva	Switzerland	Yes	2006
86	Global HIV Vaccine Enterprise	GHV	vaccineenterprise.org	6	New York City, NY	USA	Yes	2004
87	Global Hope Network International	GHN	globalhopenetwork.org	6	Geneva	Switzerland	No	1999
88	Global Network of People Living with HIV	GNP	gnpplus.net	6	Amsterdam	Netherlands	No	1986
89	Guttmacher Institute	GUI	guttmacher.org	6	New York City, NY	USA	Yes	1968
90	Health Action International	HAI	haiweb.org	6	Geneva	Switzerland	Yes	1981
91	Health Skepticism Inc	HIS	healthyskepticism.org	6	Port Willunga	Australia	Yes	1983
92	Health Volunteers Overseas	HVO	hvousa.org	6	Washington, DC	USA	Yes	1986
93	HealthCare Volunteer	HCV	healthcarevolunteer.com	6	Los Altos, CA	USA	Yes	2005
94	HealthRight International	HRI	healthright.org	6	New York City, NY	USA	Yes	1990
95	Hellen Keller International	HKI	hki.org	6	New York City, NY	USA	Yes	1915
96	Higher Education for Development	HED	hedprogram.org	6	Washington, DC	USA	No	1918
97	IBFAN (International Baby Food Action Network)	IBF	ibfan.org	6	Geneva	Switzerland	Yes	1979
98	Ibis Reproductive Health	IRH	ibisreproductivehealth.org	6	Cambridge, MA	USA	No	2002
99	ICASCO (International Council of AIDS Service Organizations)	ICA	icaso.org	6	Toronto	Canada	Yes	1991
100	Infectious Disease Research Institute	IDR	idri.org	6	Washington, DC	USA	Yes	1993
101	Institute of Food Technologists	IFT	ift.org	6	Chicago, IL	USA	No	1939
102	International AIDS Society	IAS	iasociety.org	8	Geneva	Switzerland	Yes	1988
103	International AIDS Vaccine Initiative	IAV	iavi.org	6	New York City, NY	USA	Yes	1996
104	International Association for Food Protection	IAF	foodprotection.org	6	Des Moines, IA	USA	No	1911
105	International Association of National Public Health Institutes	IAN	ianphi.org	2	Atlanta, GA	USA	Yes	2006
106	International Association of Providers of AIDS Care	IAP	iapac.org	8	Chicago, IL	USA	Yes	1995
107	International Center for Research on Women	ICR	icrw.org	6	Washington, DC	USA	No	1976
108	International Consortium for Emergency Contraception	ICE	cecinfo.org	6	New York City, NY	USA	Yes	1996
109	UNITAID (International Drug Purchase Facility)	IDP	unitaid.eu	4	Geneva	Switzerland	Yes	2006
110	International Epidemiological Association	IEA	ieaweb.org	8	Raleigh, NC	USA	No	1954
111	International Federation of Medical Students’ Associations	IFM	ifmsa.org	6	Amsterdam	Netherlands	No	1951
112	International Finance Facility for Immunisation	IFF	iffim.org	6	London	UK	Yes	2006
113	International Food Policy Research Institute	IFP	ifpri.org	6	Washington, DC	USA	No	1975
114	International Fund for Agricultural Development	IFA	ifad.org	2	Rome	Italy	No	1977
115	International Health Partnership	IHP	internationalhealthpartnership.net	4	Washington, DC	USA	Yes	2007
116	International HIV/AIDS Alliance	IHA	aidsalliance.org	6	Hove	UK	Yes	1993
117	International Life Sciences Institute	ILS	ilsi.org	6	Washington, DC	USA	No	1978
118	International Network for Rational Use of Drugs	INR	inrud.org	4	Arlington, VA	USA	No	1989
119	International Partnership for Microbicides	IPM	ipmglobal.org	4	Silver Spring, MD	USA	Yes	2002
120	International Pharmaceutical Students’ Federation	IPS	ipsf.org	6	The Hague	Netherlands	Yes	1949
121	International Planned Parenthood Federation	IPP	ippf.org	6	London	UK	No	1952
122	International Relief & Development	IRD	ird.org	6	Arlington, VA	USA	No	1998
123	International Society for Infectious Diseases	ISI	isid.org	6	Brookline, MA	USA	Yes	1986
124	International Society of Drug Bulletins	ISD	isdbweb.org	6	London	UK	No	1986
125	International Union Against Tuberculosis and Lung Disease	IUA	theunion.org	4	Paris	France	Yes	1920
126	International Union of Food Science and Technology	IUF	iufost.org	6	Oakville	Canada	No	1970
127	International Union of Nutritional Sciences	IUN	iuns.org	6	Vienna	Austria	No	1948
128	International Vaccine Institute	IVI	ivi.int	6	Seoul	Republic of Korea	Yes	1996
129	IntraHealth International	IHI	intrahealth.org	6	Chapel Hill, NC	USA	Yes	1979
130	Ipas (formerly International Pregnancy Advisory Services)	IPA	ipas.org	6	Chapel Hill, NC	USA	Yes	1973
131	Jhpiego	JHP	jhpiego.org	6	Baltimore, MD	USA	Yes	1974
132	John Snow, Inc.	JSI	jsi.com	7	Boston, MA	USA	Yes	1978
133	Johns Hopkins Bloomberg School of Public Health	JHB	jhsph.edu	9	Baltimore, MD	USA	Yes	1916
134	Joint United Nations Programme on HIV/AIDS	JUN	unaids.org	2	Geneva	Switzerland	Yes	1996
135	London School of Hygiene and Tropical Medicine	LSH	www.lshtm.ac.uk	9	London	UK	Yes	1899
136	Malaria Foundation International	MFI	malaria.org	6	Stone Mountain, GA	USA	Yes	1992
137	Malaria No More	MNM	malarianomore.org	6	New York City, NY	USA	Yes	2006
138	Management Systems International	MSN	msiworldwide.com	7	Washington, DC	USA	No	1981
139	Médecins Sans Frontières	MSF	msf.org	6	Geneva	Switzerland	Yes	1971
140	Medicines for Malaria Venture	MMV	mmv.org	4	Geneva	Switzerland	Yes	1999
141	MediSend International	MSI	medisend.org	6	Dallas, TX	USA	Yes	1999
142	Mercy Corps	MEC	mercycorps.org	6	Portland, OR	USA	No	1979
143	Millennium Challenge Corporation	MCC	mcc.gov	1	Washington, DC	USA	No	2004
144	National Institutes of Health	NIH	nih.gov	1	Bethesda, OH	USA	Yes	1887
145	Operation Rainbow	OPR	operationrainbow.org	6	Oakland, CA	USA	Yes	1978
146	Operation Smile	OPS	operationsmile.org	6	Virginia Beach, VA	USA	Yes	1982
147	Operation USA	OPU	opusa.org	6	Los Angeles, CA	USA	No	1979
148	Oxfam International	OXI	oxfam.org	6	Washington, DC	USA	No	1995
149	Pan American Society for Clinical Virology	PAS	pascv.org	8	Raleigh, NC	USA	Yes	1977
150	Pangaea Global AIDS Foundation	PGA	pangaeaglobal.org	6	Oakland, CA	USA	Yes	2001
151	Partners in Health	PIH	pih.org	6	Boston, MA	USA	Yes	1987
152	Partnership for Maternal, Newborn and Child Health	PMN	who.int/pmnch	4	Geneva	Switzerland	Yes	2005
153	PATH	PAT	path.org	6	Seattle, WA	USA	Yes	1977
154	Pathfinder International	PAI	pathfinder.org	6	Watertown, MN	USA	Yes	1957
155	Pediatric Infectious Diseases Society	PID	pids.org	8	Arlington, VA	USA	Yes	1984
156	Plan International	PLI	plan-international.org	6	Woking	UK	No	1937
157	Population Action International	PAN	populationaction.org	6	Washington, DC	USA	No	1965
158	Population Council	POC	popcouncil.org	6	New York City, NY	USA	No	1952
159	Population Media Center	PMC	populationmedia.org	6	Shelburne, MA	USA	Yes	1998
160	Population Reference Bureau	PRB	prb.org	6	Washington, DC	USA	No	1929
161	Population Services International	PSI	psi.org	6	Washington, DC	USA	Yes	1970
162	Project HOPE	PRH	projecthope.org	6	Millwood, VA	USA	Yes	1958
163	Public Health Institute	PHI	phi.org	6	Oakland, CA	USA	Yes	1964
164	RAND Corporation	RAC	rand.org	6	Santa Monica, CA	USA	No	1948
165	Refugees International	REI	refintl.org	6	Washington, DC	USA	No	1979
166	Reproductive Health Response in Crises Consortium	RHR	rhrc.org	6	Minneapolis, MN	USA	Yes	1995
167	Reproductive Health Supplies Coalition	RHS	rhsupplies.org	4	Brussels	Belgium	Yes	2004
168	Research Triangle Institute International	RTI	rti.org	6	Durham (Research Triangle Park), NC	USA	No	1958
169	Roll Back Malaria Partnership	RBM	rollbackmalaria.org	4	Geneva	Switzerland	Yes	1998
170	Sabin Vaccine Institute	SVI	sabin.org	6	Washington, DC	USA	Yes	1993
171	Save the Children International	SCI	savethechildren.net	6	London	UK	No	1919
172	Society for Public Health Education	SPH	sophe.org	8	Washington, DC	USA	Yes	1950
173	Society for Research on Nicotine and Tobacco	SRN	srnt.org	6	Madison, WI	USA	No	1994
174	Stephen Lewis Foundation	SLF	stephenlewisfoundation.org	6	Toronto	Canada	Yes	2003
175	Stop TB Partnership	STP	stoptb.org	4	Geneva	Switzerland	Yes	2001
176	Swiss Tropical and Public Health Institute	STH	swisstph.ch	1	Basel	Switzerland	Yes	1943
177	Syrian Center for Tobacco Studies	SCT	scts-sy.org	6	Aleppo	Syrian Arab Republic	No	2002
178	TB Alert	TBA	tbalert.org	6	Brighton	UK	Yes	1998
179	The Earth Institute, Columbia University	TEI	earthinstitute.columbia.edu	9	New York City, NY	USA	No	1995
180	The Global Fund to Fight AIDS, Tuberculosis and Malaria	TGF	theglobalfund.org	4	Geneva	Switzerland	Yes	2002
181	The Water Project	TWP	thewaterproject.org	6	Concord, NH	USA	No	2006
182	Tobacco Free Nurses	TFN	tobaccofreenurses.org	6	Los Angeles, CA	USA	No	2003
183	Tostan	TOS	tostan.org	6	Dakar	Senegal	No	1974
184	Treatment Action Group	TAG	treatmentactiongroup.org	6	New York City, NY	USA	Yes	1992
185	TuBerculosis Vaccine Initiative	TVI	tbvi.eu	6	Lelystad	Netherlands	Yes	2008
186	Unite for Sight	UFS	uniteforsight.org	6	New Haven, CT	USA	Yes	2000
187	United Nations Children’s Fund	UNC	unicef.org	2	New York City, NY	USA	No	1946
188	United Nations Development Programme	UND	undp.org	2	New York City, NY	USA	No	1966
189	United Nations Foundation	UNF	unfoundation.org	6	Washington, DC	USA	No	1998
190	United Nations Population Fund	UNP	unfpa.org	2	Geneva	Switzerland	No	1969
191	United States Agency for International Development	USA	usaid.gov	1	Washington, DC	USA	No	1961
192	United States Department of Health & Human Services, Office of Global Affairs	USD	globalhealth.gov	1	Washington, DC	USA	Yes	2002
193	University of California, San Francisco	UCS	www.ucsf.edu	9	San Francisco, CA	USA	Yes	1864
194	VSO (Voluntary Service Overseas)	VSO	vso.org.uk	6	Kingston upon Thames	UK	No	1958
195	Women Deliver	WOD	womendeliver.org	6	New York City, NY	USA	Yes	2007
196	World AIDS Campaign	WAC	worldaidscampaign.org	6	Cape Town	South Africa	Yes	1997
197	World Bank	WOB	worldbank.org	3	Washington, DC	USA	No	1944
198	World Food Programme	WFP	wfp.org	2	Rome	Italy	No	1961
199	World Health Organization	WHO	who.int	2	Geneva	Switzerland	Yes	1948
200	World Lung Foundation	WLF	worldlungfoundation.org	6	New York City, NY	USA	Yes	2004
201	World Vision International	WVI	wvi.org	6	Uxbridge	UK	No	1950
202	Worldwatch Institute	WOI	worldwatch.org	6	Washington, DC	USA	No	1974
203	Yale School of Public Health	YSP	publichealth.yale.edu	9	New Haven, CT	USA	Yes	1946

The numerical codes in the column labeled ‘Type’ represent the following actor types:

1 = National governments

2 = United Nations entities and intergovernmental organizations

3 = Multilateral development banks

4 = Public-private partnerships

5 = Philanthropic organizations

6 = Global civil society and non-governmental organizations

7 = Private industry

8 = Professional associations

9 = Academic institutions

#### Social network analysis

The social network included 198 nodes, representing all global health actors identified through “related:URL” searches, and 412 edges. This does not include the five additional WHO-hosted partnerships that bring the count of global health actors to 203, as they were not identified using the related search function and therefore are not a formal part of the identified online network structure. The social network showed an average of 2.081 connections per actor and a network diameter of 4. See Table 3 for rankings of the top ten global health actors by degree, betweenness centrality, and closeness centrality. Global health actors’ characteristics and centrality scores were used to explore the structure of the network.

**Table 3:** Top ten global health actors by social network metrics

Rank	Actor	Degree
1	WHO	48
2	GHO	40
3	FHI	39
4	TGF	38
5	USA	37
6	POC	34
7	MSF	31
8	PAN	29
9	CDP	23
10	UNC	23
Rank	Actor	Closeness centrality
1	WHO	1.868020305
2	TGF	2.208121827
3	GHO	2.279187817
4	FHI	2.319796954
5	USA	2.324873096
6	MSF	2.426395939
7	PAN	2.456852792
8	UNC	2.512690355
9	MFI	2.532994924
10	CDP	2.558375635
Rank	Actor	Betweenness centrality
1	WHO	2986.842403
2	GHO	607.1230453
3	TGF	557.3348557
4	CDP	488.7137265
5	MSF	485.1809524
6	USA	394.0630366
7	UNC	352.6553336
8	FHI	297.9844801
9	STP	259.9629191
10	POC	234.107373

#### Type of entity

**Fig. 2:**
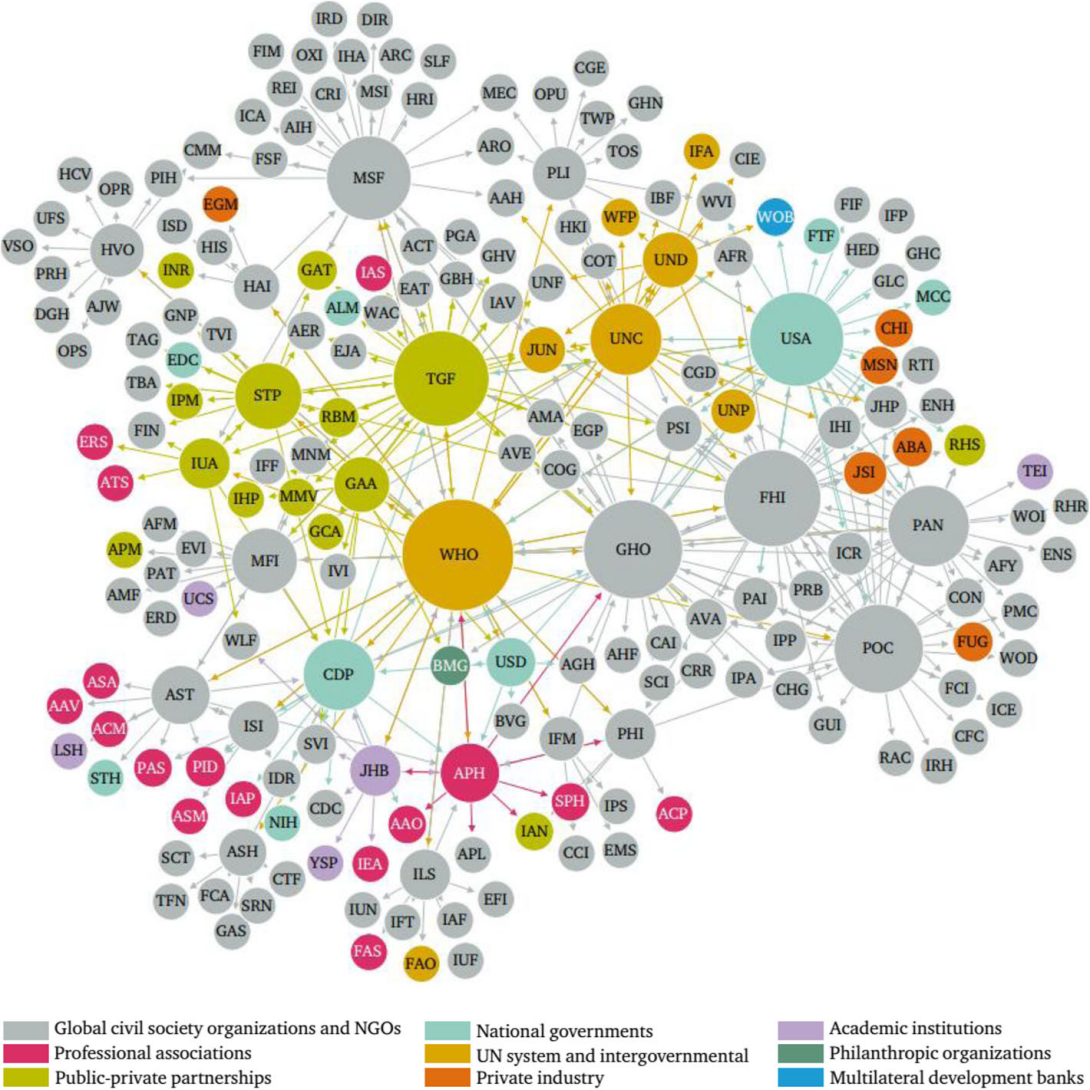
Network mapping of global health actors by type. Node size is ranked by degree; node colour is partitioned by type of actor; and edges are coloured by source node

The majority of identified global health actors were global civil society organizations and non-governmental organizations (*n* = 138), followed by public-private partnerships (*n* = 18), professional associations (*n* = 16), UN entities and intergovernmental organizations (*n* = 11), national governments (*n* = 7), private industry (*n* = 6), academic institutions (*n* = 5), multilateral development banks (n = 1) and philanthropic institutions (*n* = 1) (see Fig. 2).

#### Location

**Fig. 3:**
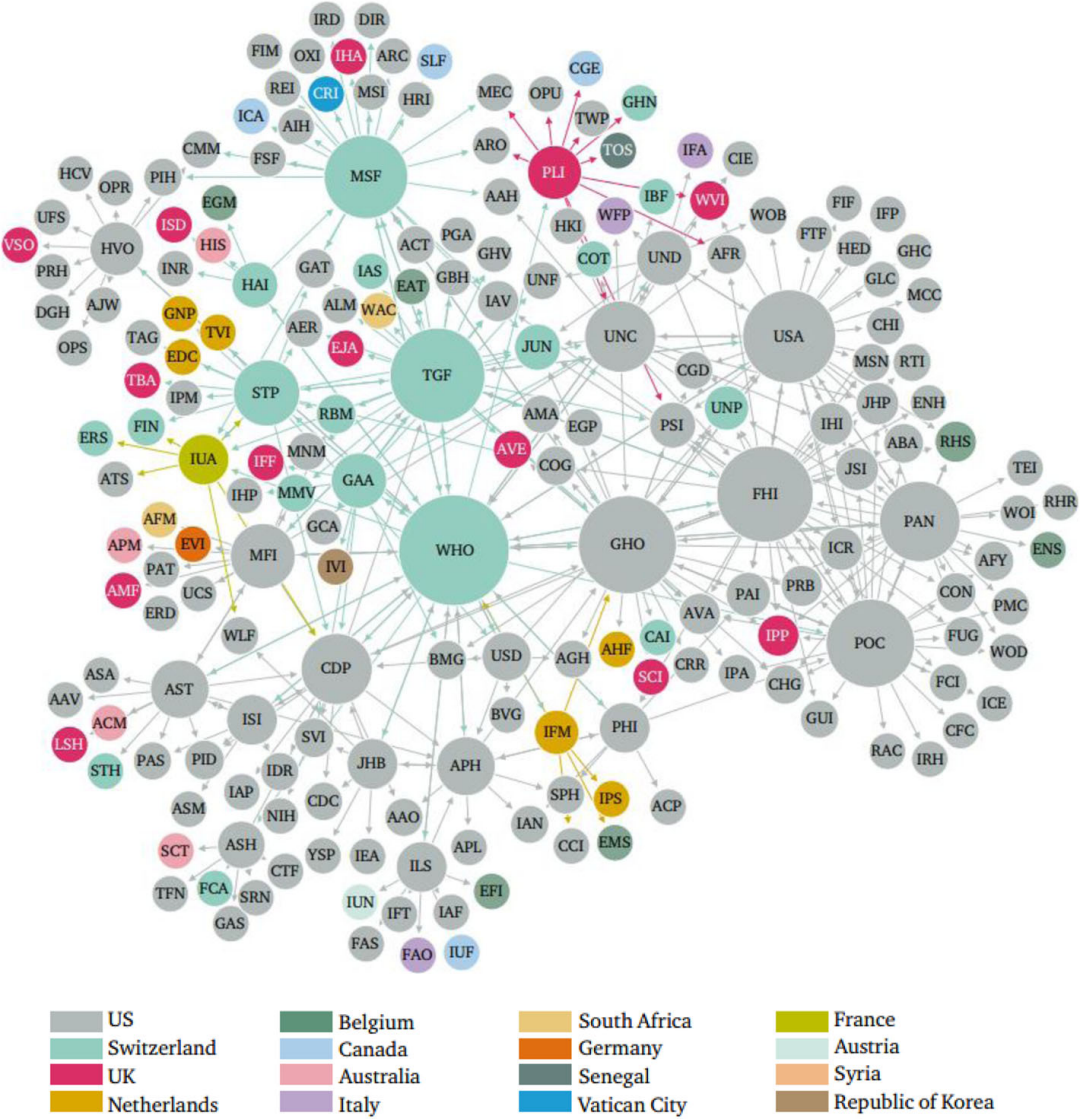
Network mapping of global health actors by country location of international headquarters. Node size is ranked by degree, node colour is partitioned by headquarters location, and edges are coloured by source node

International headquarters of the 203 global health actors were located in 16 countries and 73 cities (see Fig. 3). 98.5% of headquarters were located in high-income countries. Two actors’ headquarters were located in low- or lower-middle-income countries (i.e., Syrian Arab Republic and Senegal) and one in an upper-middle-income country (i.e., South Africa). The most common countries for global health actors to headquarter themselves were the U.S. (*n* = 135), Switzerland (*n* = 23), and the United Kingdom (*n* = 13), followed by Belgium (*n* = 7), The Netherlands (*n* = 6), and Canada (*n* = 4). The top three most common cities for headquarters were Washington, D.C. (*n* = 42), New York City (*n* = 28), and Geneva (*n* = 21).

#### Year of inception

**Fig. 4:**
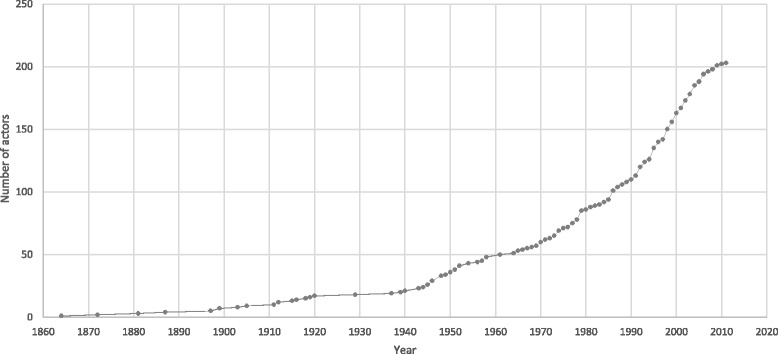
Rate of inception of global health actors over time

**Fig. 5:**
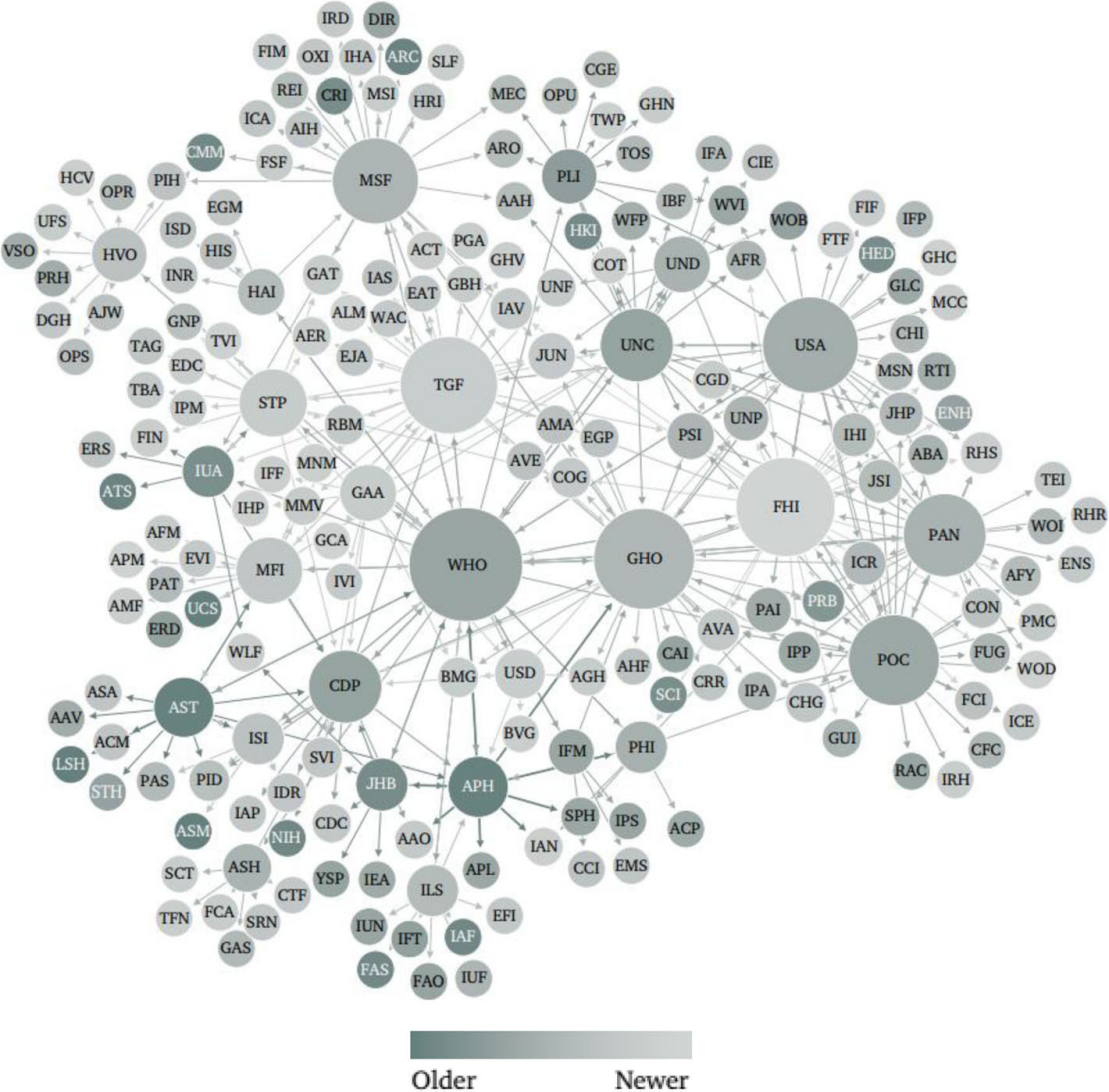
Network mapping of global health actors by year of inception. Node size is ranked by degree; node colour is ranked by year of inception, where darker tones indicate an earlier year of inception and lighter ones indicate newer actors

Identified global health actors were created between 1864 and 2011, with the rate of inception of actors over time displayed in Fig. 4 and the inception of actors over time displayed as a network in Fig. 5.

#### Primary intent

**Fig. 6:**
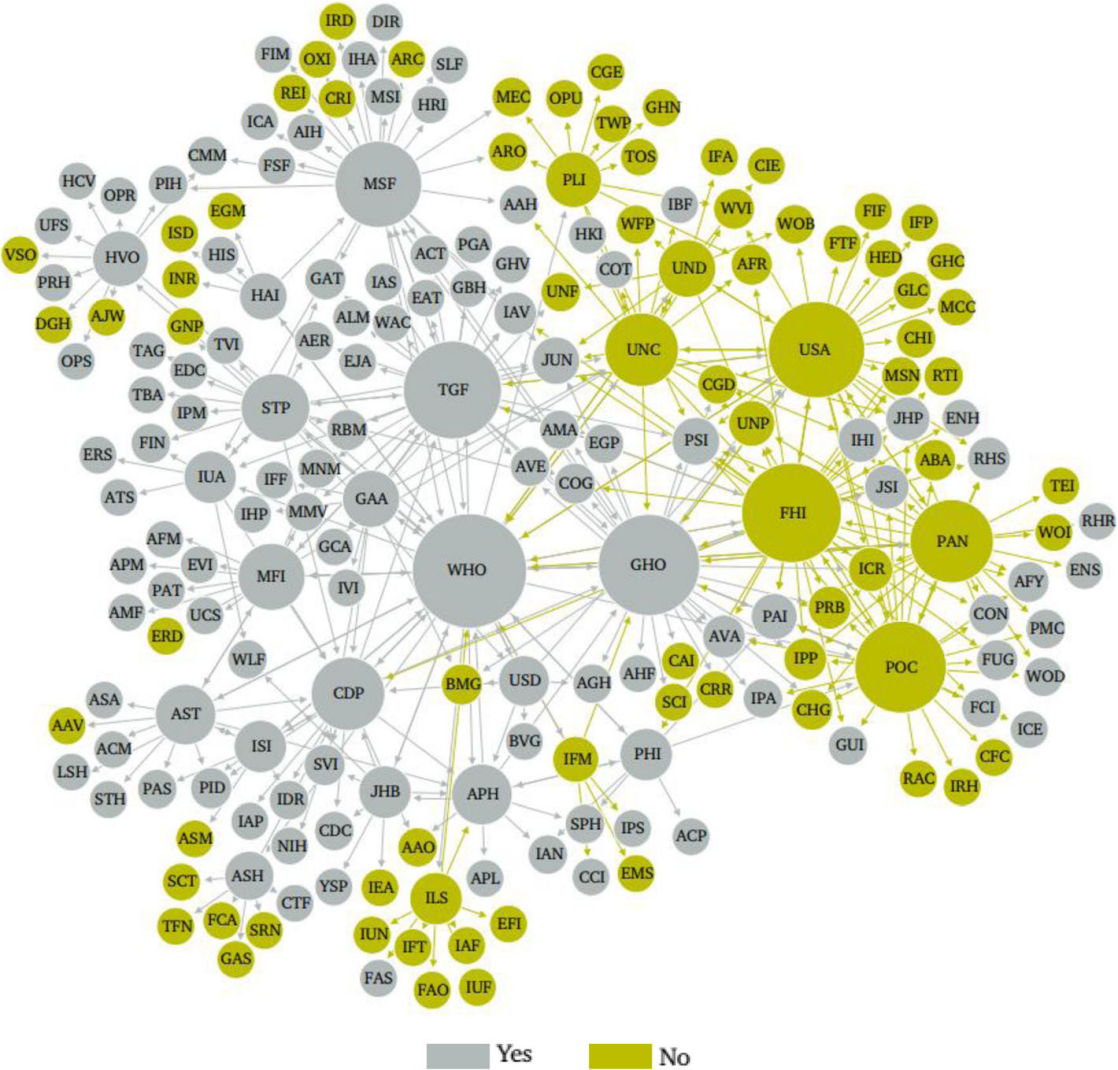
Network mapping of global health actors by intent to improve health. Node size is ranked by degree; node colour is partitioned by whether or not improving health is the primary intent of the organization; and edges are coloured by source node

61.6% of global health actors (*n* = 125) listed improving health as the sole primary intent of their organization, compared to 38.4% of actors (*n* = 78) who listed improving health as one of multiple primary intents (see Fig. 6).

### Validation exercise

The key global health system stakeholders who were consulted on the study’s findings agreed that the definition developed for the global health system was analytically helpful. To their knowledge, the research was noted to be the first systematic attempt to map the global health system, and that having access to the basic list of actors was helpful. However, the participants agreed the resulting map of 203 actors did not comprehensively present the most important and influential actors in the global health system. The exercise revealed that senior leaders at major global health organizations value global representativeness, and that the results of future studies would be more helpful if their findings were generalizable at the global level.

## Discussion

### Principal findings

Our definition of the global health system was used effectively in a novel search methodology that took advantage of one of the world’s most powerful Internet crawlers and related search algorithms (i.e., Google “related:URL” search). The methodology proved useful and efficient, systematically generating the largest network of global health actors to date. Notably, all identified actors were organizations. However, the network did include organizations founded by individuals, such as the Bill & Melinda Gates Foundation, Elizabeth Glaser Pediatric AIDS Foundation, and Elton John AIDS Foundation. This reflects the tendency for websites to be created by organizations, whereas individuals tend to use social media or web pages on organizations’ websites for their online presence (e.g., Facebook pages and Twitter accounts representing celebrities or university webpages featuring faculty members).

Basic analysis of the network structure reveals interesting findings about the online network of global health actors that may shed light on the offline global health system structure. The 26 global health actors identified in the search “related:www.who.int”, which subsequent searches were based upon, are almost identical to the top 26 global health actors by degree centrality (after WHO, which ranks first). The only exception was the International Federation of Medical Students’ Associations, which was identified in the first related search but had one too few connections to rank in the top 27 actors by degree. Interestingly, Population Services International and UNAIDS – two organizations that were not identified in the first related search and therefore whose placements in the network were not dictated by the search methodology – tied for the 27th ranking by degree. This indicates that the related search function found them to be highly related to many other websites. They are placed in relatively central position in the online network in terms of activity. Offline, this may suggest Population Services International and UNAIDS are relatively well-connected and active players in the global health system.

Within the group of 26 actors identified through the first related search, there were eight organizations that consistently ranked in the top ten global health actors after WHO by degree, betweenness centrality, and closeness centrality: FHI360, Global Fund to Fight AIDS, Tuberculosis & Malaria, Global Health Council, Médecins Sans Frontières, Stop TB Partnership, U.S. Agency for International Development, U.S. Centers for Disease Control and Prevention, and UNICEF. This result indicates the websites of these actors are central to the network: they are related to a relatively large number of other actors’ websites, probably important in facilitating connections between websites, and allow for efficient connections to other actors in the system.

Through basic characterization of global health actors, various conclusions can be drawn. Categorization of global health actors by type indicates the overwhelming presence of global civil society organizations and non-governmental organizations in the online network of global health actors. The emergence of global health public-private partnerships is seen through an online presence that makes up just over 9% of identified global health actors [3]. Despite overwhelming influence of actors like the Bill & Melinda Gates Foundation, [21] philanthropic organizations represented only 0.5% of identified global health actors. Visualization of the network by type of actor shows actors of the same type in distinct groupings, such as those of public-private partnerships, UN entities and intergovernmental organizations, and private industry respectively, suggesting that websites of the same type of global health actors are related to one another. Interestingly, the pre-chosen focal point of the global health system – the WHO – is directly connected to all actor types with the exception of philanthropic organizations and multilateral development banks, for which only one actor was identified in each category.

Geographical distribution of actors’ international headquarters, spanning 73 cities across 16 countries, points to the global nature of the system. However, the overwhelming presence of actors’ headquarters in high-income countries – more than half in the U.S. – clearly suggests an uneven distribution of actors’ leadership globally. Significantly, no actor websites were located in the BRIC countries (i.e., Brazil, Russia, India and China). Several major centres of activity were identified, notably Washington, D.C., New York City, and Geneva. This reflects the location of various influential actors – global health and non-global health alike – around which global health actors have decided to co-locate. For example, the U.S. government and World Bank are headquartered in Washington, D.C., the UN is based in New York City, and WHO is in Geneva.

The inception of new global health actors over time indicates three distinct phases of rapid growth. The rate of inception of global health actors remained low until 1945, when a period of increased growth began, continuing until 1952. This growth coincides with the creation of the UN system in 1945 [22]. New global health actors may have been created as part of the nascent UN system itself, or in tandem with its development and also with that of other important multilateral organizations. A second phase of expansion began in 1970 and lasts through the decade, with a peak occurring in 1979. This expansion may reflect increased interest in international economic development at that time. For example, in 1973 the World Bank announced its pledge to increase financing for development by 40% over the following 5 years [23]. Last, an increase in actors is seen beginning in 1986 and continuing until 2006, coinciding with a quintupling of global health financing [24]. Each subsequent surge in the creation of new actors occurs at a higher rate than the previous one: an average of 2.00 actors were established per year from 1945 to 1952, 2.55 per year from 1970 to 1979, and 4.91 per year from 1986 to 2006). This reflects an increase in the rate of growth of the global health system over time. The period of analysis ends with a lull in inception of new actors, due in part to the decade being incomplete, but also probably due to how emerging actors’ websites may need time to gain relevance and popularity online and the 2008 global financial crisis.

Visualization of the network by year of inception shows newer global health actors in more central positions, and older organizations on the periphery of the network, indicating that the most influential actors in the global health system may not necessarily be the most established ones.

The proportion of actors whose primary intent is to improve health (61.6%), versus those who describe improving health as one of their primary intents (38.4%), illustrates the complex interaction between health and other global policy domains such as economic development and environmental protection. Network visualization shows some trends according to primary intent, such as in the high number of organizations for which improving health was not the primary intent that are connected to the U.S. Agency for International Development and the International Life Sciences Institute, respectively – two organizations for which improving health was not the sole primary intent.

### Main strengths and limitations

We developed a clear, comprehensive and practical definition for the global health system that has the potential to facilitate consistent and in-depth research about this system in the future. In our study, the definition proved operational to map the structure of this system and analytically helpful for facilitating further understanding of the group of actors and their interconnections that people have come to naturally think of as the ‘global health system’. While this study was designed to yield a map of actors, it did not select actors based on their power or influence; this means the results are limited in terms of what features are analyzed. Results may also overlook some of the most important entities. The validation exercise with key global health stakeholders in December 2014 confirmed these findings; stakeholders agreed the definition was analytically helpful and the consultation revealed that the derived mapping does not exactly match with prevailing expert views of the system. However, this article presents a first systematic mapping of the global health system that can be improved upon in future exercises. Importantly, the use of our definition combined with Internet searches allowed for the systematic compilation of the largest list of global health actors to date. While the algorithm underlying the Google query refinement “related:URL” is not publicly available, the search engine is publicly available which makes it possible to replicate the methodology. Notably, any replications of the methodology will yield results that reflect the relationship between web pages on the date it is carried out.

Interestingly, despite defining the global health system from a holistic, global perspective, results may suggest a global health system characterized predominantly by the development agenda. This may reflect the nature of the global health system as it currently stands, defined by years of responding to global health challenges through a development lens. Alternatively, the methodology may have captured a portion of the global health system that is disproportionately focused on development assistance, perhaps as a result of using WHO as the search’s starting point. This could have occurred due to a tendency for WHO to provide links to its funders on its website. In the latter case, the post-2008 emergence of global health actors may have been underestimated, as these actors may be more likely to focus on global public goods and threats rather than development assistance to low- or middle-income countries. In this case, the search algorithm may not have found them to be related to actors identified earlier in the search.

By placing WHO at the centre of the system, this study used only one point of entry to probe the global health system. Using WHO’s website as the starting point of the search may have biased the search in favour of those types of actors to which WHO’s internal policies allow the organization to hyperlink. This may have increased the number of traditional and non-controversial actors identified, such as other UN entities and major WHO funders. However, our decision to start with WHO is justified by the widely acknowledged prominence and important role of this UN entity within the global health system. Other points of entry in approaching this exercise have their own advantages and disadvantages; for example, we could have begun the search with several global health actors – perhaps those with the largest global health budgets – but any starting list would have created its own biases.

Two other sources of potential bias have been identified. The use of www.google.com as well as a proxy server located in the U.S. may have biased results towards actors located in the U.S. However, the U.S.-based version of Google was selected because this is the domain of the original search engine launched by Google and is thus considered its standard product on which other location-based Google search engines are based [25]. Also, the default language of the WHO’s website is English. Since related search functions consider text-based analysis of websites’ content as one input when determining if web pages are related, the initial search may have biased results against actors whose default websites are not in English. This would include many important bilateral development agencies (e.g., Norway’s Norad) and global health actors in developing countries (e.g., Brazil’s Fiocruz).

## Conclusions

This study developed a new definition for the ‘global health system’, presented a novel methodology for populating it, and began to analyze the structure of the resulting system as well as the characteristics of its components. Consultation with senior leaders from seven key global health organizations confirmed the usefulness of this exercise and revealed a need for future mapping exercises to be more globally representative.

Future research should build on the experience of this study, finding ways to more thoroughly saturate the network of actors in the global health system. For example, future approaches may involve adapting the study’s methodology by using multiple purposively selected organizations from a variety of countries, and initiating a related search using the national Google websites corresponding to their headquarters’ locations. Internet-based findings could also be triangulated with information from political and expert opinion leaders in global health. This approach may yield more geographically neutral results as well as a list of actors that more comprehensively represents the offline global health system. Furthermore, study of the system’s structure using social network analysis suggests promise for more in-depth research in this area. Future studies should explore the nature of online relationships between global health actors, in addition to studying the relationship between observed online network dynamics and actual roles and influence of actors in the global health system.

Ultimately, future research should strive towards an empirically-derived mapping of the global health system that is representative of the real-world network and can be updated frequently or perhaps even in real-time. This is a critical starting point to facilitate more in-depth analysis of the global health system, including exploration of how and how well global health actors operate within the system’s governance, finance, and delivery arrangements, and the impact of related global policy domains on the system’s functions. Such directions for future research are important to increase our understanding of which actors are undertaking what efforts in global health and what shapes these interactions, allowing better coordination of activities in hopes of achieving the world’s health goals.

## Additional files

Additional file 1: Literature review. (DOCX 29 kb)

Additional file 2: Pilot search. (DOCX 14 kb)

Additional file 3: Title and abstract screening form. (DOCX 14 kb)

Additional file 4: Website screening form. (DOCX 16 kb)
