# Supplementary material for: Defining the global health system and systematically mapping its network of actors
Source: Global Health. 2018 Apr 17;14:38. doi: 10.1186/s12992-018-0340-2 (PMC5904998; doi:10.1186/s12992-018-0340-2)
Supplement: Supplementary file 1 — Literature review. (DOCX 29 kb) [file 12992_2018_340_MOESM1_ESM.docx]

**Additional file 1**

**WEB APPENDIX A: LITERATURE REVIEW**

The literature review methodology was based on the methodology described in: *Marchal, B., Cavalli, A., & Kegels, G. (2009). Global health actors claim to support health system strengthening—is this reality or rhetoric? PLoS medicine, 6(4), e1000059.* This includes following a framework to search for articles, and scanning titles and abstracts to select relevant papers. Following title and abstract screening, selected papers were read to extract definitions for relevant terms such as the global health system, the health system, and the system for global health governance. The list of terms of interest was flexible. The search methodology is outlined in steps below, followed by corresponding lists of literature consulted.

1. At the time of the literature review, the source of the commonly cited statistic that there are more than 40 bilateral donors, 26 UN agencies, 20 global and regional funds and 90 global health initiatives in the global health system was attributed to: *McColl, K. (2008). Europe Told to Deliver More Aid for Health. The Lancet, 371(9630): 2072-2073.* Accordingly, PubMed and Google Scholar were searched for the number of citations made to McColl (2008). The statistic was later sourced to the U.K. Department for International Development’s 2007 press release, “The international health partnership launched today,” cited in the main text of the article.

From the list of citations, scholarly articles were read to determine the nature of the reference to McColl (2008). If the citation was considered relevant, the entire article was read to extract relevant definitions. Search results were as follows:

- Barnett, T., & Sorenson, C. (2011). Infectious disease surveillance in the United States and the United Kingdom: from public goods to the challenges of new technologies. *Journal of health politics, policy and law*, *36*(1), 165-185.
- Ciccone, D. K. (2010). Arguing for a centralized coordination solution to the public-private partnership explosion in global health. *Global health promotion*,*17*(2), 48-51.
- Feldbaum, H., Lee, K., & Michaud, J. (2010). Global health and foreign policy.*Epidemiologic reviews*, *32*(1), 82-92.*
- Fidler, D. P. (2010). *The challenges of global health governance*. Council on Foreign Relations, Incorporated.
- Frenk, J., & Moon, S. (2013). Governance challenges in global health. *New England Journal of Medicine*, *368*(10), 936-942.*
- Gill, R. (2011). Chapter 7: World Development Organization. In S. Hoffman (Ed.), *STUDENT VOICES 2: Assessing Proposals for Global Health Governance Reform* (pp. 92–103). Hamilton: McMaster Health Forum.
- Gostin, L. O. (2012). A framework convention on global health: health for all, justice for all. *JAMA*, *307*(19), 2087-2092.*
- Harries, A. D., Jensen, P. M., Zachariah, R., Rusen, I. D., & Enarson, D. A. (2009). How health systems in sub-Saharan Africa can benefit from tuberculosis and other infectious disease programmes [Unresolved issues]. *The International Journal of Tuberculosis and Lung Disease*, *13*(10), 1194-1199.
- Lankenau, B. H., & Stefan, M. D. (2013). Public Health, NCDs, Health Promotion, and Business Partnering: Benefits, Concerns, Remedies, and Moving Towards Creative Partnering. In *Global Handbook on Noncommunicable Diseases and Health Promotion* (pp. 345-363). Springer New York.
- Schneider, K., & Garrett, L. (2009). The end of the era of generosity? Global health amid economic crisis. *Philosophy, Ethics, and Humanities in Medicine*,*4*(1), 1.
- Sridhar, D. (2009). Post-Accra: is there space for country ownership in global health?. *Third World Quarterly*, *30*(7), 1363-1377.*
- Sridhar, D., Khagram, S., & Pang, T. (2008). Are existing governance structures equipped to deal with today’s global health challenges-Towards systematic coherence in scaling up. *GLOBAL HEALTH*, *2*(2).*
- Sridhar, D. (2010). Seven challenges in international development assistance for health and ways forward. *The Journal of Law, Medicine & Ethics*, *38*(3), 459-469.*
- Youde, J. (2012). *Global health governance*. Polity.

2. Google Scholar was searched using the query “global health system”. Titles and descriptions were screened for the first ten pages of results, and useful pages were identified. Search results were as follows:

- Benatar, S. R., Daar, A. S., & Singer, P. A. (2003). Global health ethics: the rationale for mutual caring. *International Affairs*, *79*(1), 107-138.
- Frenk, J. (2010). The global health system: strengthening national health systems as the next step for global progress. *PLoS medicine*, *7*(1), e1000089.*
- Jong-Wook, L. (2003). Global health improvement and WHO: shaping the future. *The Lancet*, *362*(9401), 2083-2088.
- Keusch, G. T., Kilama, W. L., Moon, S., Szlezak, N. A., & Michaud, C. M. (2010). The global health system: linking knowledge with action—learning from malaria. *PLoS medicine*, *7*(1), e1000179.*
- Lopez, A. D., Mathers, C. D., Ezzati, M., Jamison, D. T., & Murray, C. J. (2006). Global and regional burden of disease and risk factors, 2001: systematic analysis of population health data. *The Lancet*, *367*(9524), 1747-1757.
- Magnussen, L., Ehiri, J., & Jolly, P. (2004). Comprehensive versus selective primary health care: lessons for global health policy. *Health affairs*, *23*(3), 167-176.
- Marchal, B., Cavalli, A., & Kegels, G. (2009). Global health actors claim to support health system strengthening—is this reality or rhetoric?. *PLoS medicine*, *6*(4), e1000059.*
  - - Supporting information file 1: Methodology
    - Supporting information file 2: A summary of policies and interventions of global health actors in the domain of health system strengthening
- McCannon, C. J., Berwick, D. M., & Massoud, M. R. (2007). The science of large-scale change in global health. *Jama*, *298*(16), 1937-1939.
- Moon, S., Szlezák, N. A., Michaud, C. M., Jamison, D. T., Keusch, G. T., Clark, W. C., & Bloom, B. R. (2010). The global health system: lessons for a stronger institutional framework. *PLoS medicine*, *7*(1), e1000193.*
- Pogge, T. W. (2005). Human rights and global health: a research program.*Metaphilosophy*, *36*(1‐2), 182-209.
- Ruger, J. P. (2007). Global health governance and the World Bank. *Lancet*,*370*(9597), 1471.
- Szlezák, N. A., Bloom, B. R., Jamison, D. T., Keusch, G. T., Michaud, C. M., Moon, S., & Clark, W. C. (2010). The global health system: actors, norms, and expectations in transition. *PLoS Medicine*, *7*(1), e1000183.*
- Villar, J., Valladares, E., Wojdyla, D., Zavaleta, N., Carroli, G., Velazco, A., ... & Acosta, A. (2006). Caesarean delivery rates and pregnancy outcomes: the 2005 WHO global survey on maternal and perinatal health in Latin America. *The Lancet*, *367*(9525), 1819-1829.
- Wootton, R., Youngberry, K., Swinfen, P., & Swinfen, R. (2004). Prospective case review of a global e-health system for doctors in developing countries.*Journal of telemedicine and telecare*, *10*(suppl 1), 94-96.
- World Health Organization. (2007). Everybody's business--strengthening health systems to improve health outcomes: WHO's framework for action.*

3. Some articles were identified through “snowballing”, in which relevant articles cited in a search result were accessed for further information. This includes:

- Dodgson, R., Lee, K., & Drager, N. (2002). Global health governance. *A Conceptual Review, London/Geneva*.*
- World Health Organization. (2000). World Health Report. Geneva: World Health Organization.*

4. Supplementary searches were conducted using Google Scholar to gain a broader understanding of how global systems, focusing on fields of security, law, and governance, and their interactions with health, are defined (e.g. the global health security regime, global health cooperation). Based on recommendation from SJH, relevant articles written by Simon Rushton and Kelley Lee were identified, specifically through a review of Google Scholar search results (first 20 results each). In addition, searches were conducted for “global health law” (first 50 results), “global health law system” (first 20 results), “global health politics” (first 50 results), and global health politics system (first 20 results). Last, an article written by SJH on the global health security regime was consulted. The following articles were selected:

- Bettcher, D., & Lee, K. (2002). Globalisation and public health. *Journal of Epidemiology and Community Health*, *56*(1), 8-17.*
- Feldbaum, H., Lee, K., & Michaud, J. (2010). Global health and foreign policy. *Epidemiologic reviews*, *32*(1), 82-92.
- Fidler, D. P. (2009). After the revolution: global health politics in a time of economic crisis and threatening future trends. *2 Global Health Governance (2008/09)*.
- Gostin, L. O., & Mok, E. A. (2009). Grand challenges in global health governance. *British Medical Bulletin*, *90*(1), 7-18.*
- Gostin, L. O., & Taylor, A. L. (2008). Global health law: a definition and grand challenges. *Public Health Ethics*, *1*(1), 53-63.*
- Hoffman, S. J. (2010). The evolution, etiology and eventualities of the global health security regime. *Health policy and planning*, *25*(6), 510-522.*
- Lee, K., Sridhar, D., & Patel, M. (2009). Bridging the divide: global governance of trade and health. *The Lancet*, *373*(9661), 416-422.*
- McInnes, C., & Lee, K. (2006). Health, security and foreign policy. *Review of International Studies*, *32*(01), 5-23.*
- Ruger, J. P. (2008). Normative foundations of global health law. *The Georgetown law journal*, *96*(2), 423.*
- Rushton, S. (2010). AIDS and international security in the United Nations System. *Health policy and planning*, *25*(6), 495-504.
- Rushton, S. (2011). Global Health Security: Security for Whom? Security from What?. *Political Studies*, *59*(4), 779-796.*
- Williams, O. D., & Rushton, S. (2011). Are the ‘Good Times’ over? Looking to the future of global health governance. *Global health governance*, *1*, 1-15.*

*Indicates article was selected and relevant definitions extracted.
