# Supplementary material for: Defining the global health system and systematically mapping its network of actors
Source: Global Health. 2018 Apr 17;14:38. doi: 10.1186/s12992-018-0340-2 (PMC5904998; doi:10.1186/s12992-018-0340-2)
Supplement: Supplementary file 2 — Pilot search. (DOCX 14 kb) [file 12992_2018_340_MOESM2_ESM.docx]

**Additional file 2: PILOT SEARCH**

A pilot search to test the network mapping methodology was conducted on January 28, 2014. Nineteen website home pages for "related:" searches were identified from a list of example global health actors found in Table 1 of the article: *Frenk J, Moon S. Governance challenges in global health. N Engl J Med. 2013 Mar 7 [cited 2014 Jul 12];368(10):936–42. Available from: http://www.ncbi.nlm.nih.gov/pubmed/23465103*. Searches were conducted using Google, each search yielding between 8 and 50 results. Overall, these pilot searches yielded 617 total and 497 unique URL results, which were exported from Google. Twenty of 497 unique URL results were selected using a random number generator to test and refine inclusion/exclusion criteria.

Google searches were conducted for each URL and resulting titles and abstracts were evaluated conservatively as to whether or not they met the definition for a global health actor. Fifteen of 20 URLs somewhat or entirely met inclusion/exclusion criteria. These URLs were accessed for their websites' "About" page, and information was evaluated using three criteria according to the definition of a global health actor, which are 1) The result represents an individual or organization (i.e., an actor); 2) The actor operates in three or more countries (e.g. transnationally), and 3) The actor demonstrates a primary intent to improve health. Eight of 15 results were included in the final list of global health actors, indicating 40% specificity of the search methodology. "About" pages of initially excluded URLs were accessed for further review, and 0 of 5 met criteria to be included as a global health actor.
