# Supplementary material for: Defining the global health system and systematically mapping its network of actors
Source: Global Health. 2018 Apr 17;14:38. doi: 10.1186/s12992-018-0340-2 (PMC5904998; doi:10.1186/s12992-018-0340-2)
Supplement: Supplementary file 3 — Title and abstract screening form. (DOCX 14 kb) [file 12992_2018_340_MOESM3_ESM.docx]

**Additional file 3: TITLE AND ABSTRACT SCREENING FORM**

**Screener Initials __________**

**General Information**

| Google search query: |  |
| --- | --- |
| Date of Title and Abstract extraction: |  |
| URL: |  |
| Title: |  |
| Abstract: |  |

**Eligibility**

| **Does the result appear to meet two of the three following criteria?** (See Appendix D for more detailed descriptions of criteria.)   1. **Does the result refer to an individual or organization?**   - Individual related keywords, e.g. workers, leaders  - Organization related keywords, e.g. center, foundation, institute, program   1. **Does the actor operate in three or more countries?**   - Related keywords, e.g. global, population, international, world, developing countries   1. **Does the actor identify improving health as one of their primary intents?**  - Health related keywords, e.g., health, HIV/AIDS, malaria, contraception, poverty, lives, death, well-being, vaccination - Health improvement related keywords, e.g. research, diplomacy, advocate, philanthropy, fundraising, grants, delivery, improve, prevention, supplies, give, stewardship, services | **Yes** _______  **No** _______ **Uncertain** _______ |
| --- | --- |
| **Note here** if abstract includes errors that make its evaluation impossible. Conduct Google search using the specific URL as the search query and review new abstract according to Appendix C. | **Searched Google for specific URL _______** |

Follow URL and access website ‘About’ page if the answer is **YES**. Continue to **Appendix D.**

**ACCESS WEBSITE: Yes** _______  **No** _______

NB: Keywords identified in Appendix C are based on learning from a pilot search. Eligible keywords are not limited to the lists above, however, should be used as guidance to identify related words.
