# Supplementary material for: Defining the global health system and systematically mapping its network of actors
Source: Global Health. 2018 Apr 17;14:38. doi: 10.1186/s12992-018-0340-2 (PMC5904998; doi:10.1186/s12992-018-0340-2)
Supplement: Supplementary file 4 — Website screening form. (DOCX 16 kb) [file 12992_2018_340_MOESM4_ESM.docx]

**Additional file 4: WEBSITE SCREENING FORM**

**Screener Initials __________**

**General Information**

| URL: |  |
| --- | --- |
| Website title: |  |
| Organization name: |  |
| About page URL:  e.g. ‘About us’, ‘Who we are’  When multiple main information pages are present, choose the most prominent one (according to which list is presented first or provides general information about the website. For about pages with multiple sections, if the main page does not provide sufficient information please access the most appropriate link available, i.e., “Overview” |  |

**Eligibility**

| **1. Actor:** Is the result an individual or an organization?  Key indicators: Site represents a specific individual or organization. Actors do not includes services, journals, or information networks/websites. Include actors like networks and coalitions if defined as organizations or if they have clear functions as an independent entity. | **Yes** _______  **No** _______ **Uncertain** _______ |
| --- | --- |
| **2. Transnational:** Does the actor operate in three or more countries?  Key indicators: Page references operations in three or more specific countries or in multiple countries, or references global or international operations more broadly. Include actors with international membership if this clearly suggests actor’s operations in multiple countries. | **Yes** _______  **No** _______ **Uncertain** _______ |
| **3. A primary intent to improve health:** Does the actor identify improving health as one of their primary intents?  Key indicators: Page identifies improving health as one of the actor’s main goals, generally, via a specific disease (e.g. HIV/AIDS) and/or sub-population (e.g. children’s health), or by particular actions (e.g. contributing to health education). Actors indicating a primary intent to improve lives and have main programs in health are eligible. | **Yes** _______  **No** _______ **Uncertain** _______ |
| **Note** **here** if the page mentions the actor is part of a ‘parent’ organization. Review ‘parent’ organization’s website for data abstraction eligibility according to Appendix C. If parent qualifies for inclusion, exclude any corresponding actor that was originally eligible (i.e., exclude children where parents are included). | ‘**Parent’ organization name _________________** |

Eligible for data abstraction if, and only if, the answer is **YES** to all three questions. If discrepancies exist, access other web pages from website to supplement initial data. Begin by accessing other web pages on “About” section, moving next to “Programs” section, and finally, to other pages on the website as necessary. Record URLs accessed and review eligibility according to Appendix D. Review any discrepancies on eligibility that cannot be resolved by discussing with the project arbitrator (Steven Hoffman).

**WEBSITE IS ELIGIBLE FOR DATA EXTRACTION: Yes** _______  **No** _______
